# Epiregulin drives keratinocyte hyperproliferation in sorafenib-induced hand-foot skin reaction: A mechanistic and therapeutic insight

**DOI:** 10.1016/j.clinsp.2025.100809

**Published:** 2025-10-31

**Authors:** Yuxin Liang, Xiao Hong, Xiaorong Zhong, Ke Chen, Jie Wang, Jingbin Zhao, Zhao Li, Jianlin Wu, Guojun Zhou, Xiaolun Huang, Zhengwei Leng

**Affiliations:** a Liver Transplantation Center and HBP Surgery, Sichuan Cancer Hospital & Institute, Sichuan Cancer Center, School of Medicine, University of Electronic Science and Technology of China, Chengdu, China; b Institute of Hepatobiliary and Pancreatic Diseases, North Sichuan Medical College, Nanchong, China

**Keywords:** Sorafenib, Hand-foot skin reaction, Epiregulin, Keratinocyte, EGFR

## Abstract

**Purpose:**

Sorafenib-induced Hand-Foot Skin Reaction (HFSR) significantly impairs patient quality of life, yet its underlying mechanisms remain poorly understood. This study investigates the pivotal role of Epiregulin (EREG), an Epidermal Growth Factor Receptor (EGFR) ligand, in driving keratinocyte hyperproliferation and the pathogenesis of HFSR.

**Methods:**

The authors established a murine HFSR model using oral sorafenib (100 mg/kg) and analyzed paw tissues for histopathological changes (H&E staining) and EREG expression (immunohistochemistry). In vitro, HaCaT keratinocytes were treated with sorafenib and recombinant EREG to assess proliferation (CCK-8 assay).

**Results:**

Sorafenib-treated mice exhibited hallmark HFSR features, including erythema, neutrophil infiltration, and thickened keratin layers (*p* < 0.001). EREG expression was markedly upregulated in affected tissues (*p* < 0.001). Notably, recombinant EREG dose-dependently enhanced HaCaT cell proliferation (*p* < 0.05), while sorafenib alone showed no direct proliferative effect.

**Conclusion:**

The present findings identify EREG as a critical mediator of keratinocyte hyperproliferation in sorafenib-induced HFSR, offering a novel therapeutic target. Moreover, EREG expression may serve as a predictive biomarker for HFSR severity, guiding personalized prophylaxis. Clinical exploration of EREG inhibition could provide a tailored approach to alleviate HFSR, improving patient adherence and treatment outcomes.

## Highlights

• First evidence linking EREG to sorafenib-induced HFSR via keratinocyte proliferation.
• Mouse model recapitulates human HFSR with neutrophil-rich inflammation and hyperkeratosis.
• EREG promotes HaCaT proliferation (*p* < 0.05), while sorafenib acts indirectly.
• 100 mg/kg sorafenib in mice (≈10 mg/kg human dose) validated by FDA scaling.
• Therapeutic potential: EREG blockade may mitigate HFSR severity.

## Introduction

Sorafenib, a multi-target Tyrosine Kinase Inhibitor (TKI), has been used in various systemic cancer treatments, including Hepatocellular Carcinoma (HCC) and Renal Cell Cancer (RCC).^1^ Notably, the approval of sorafenib by the U.S. Food and Drug Administration (FDA) represented a major advancement in managing advanced HCC by substantially improving patient survival outcomes.^2^ As recommended by multiple international clinical guidelines, sorafenib remains a standard therapeutic option for patients with advanced HCC and RCC.^3^, ^4^, ^5^

Despite its therapeutic benefits, sorafenib therapy is frequently associated with several adverse effects, among which Hand-Foot Skin Reaction (HFSR) is one of the most common and challenging complications.^6^ Clinically, HFSR is characterized by painful erythema, edema, and desquamation of the palms and soles, with affected areas typically exhibiting skin thickening and hyperkeratosis after several weeks of treatment.^7^^,^^8^ The incidence of HFSR varies between 30 % and 40 %, with approximately 8 % to 9 % of patients experiencing severe symptoms (grades 3‒4).^9^^,^^10^ These complications often force dose reduction or even discontinuation of sorafenib therapy, thereby reducing treatment efficacy and potentially accelerating disease progression.

Although the clinical manifestations of sorafenib-induced HFSR are well documented, the underlying molecular mechanisms remain incompletely understood. In particular, the potential role of Epidermal Growth Factor Receptor (EGFR) ligands, including Epiregulin (EREG), has not been fully elucidated. EREG, primarily expressed in keratinocytes and tissue-resident macrophages, plays a key role in maintaining skin homeostasis, regulating inflammation, and influencing various malignancies.^11^, ^12^, ^13^ Given the pathological features of HFSR include abnormal epidermal proliferation and structural alterations,^14^ the authors hypothesize that elevated EREG may drive keratinocyte hyperproliferation, contributing to the pathogenesis of sorafenib-induced HFSR.

Therefore, this study is designed to investigate the role of EREG in the pathogenesis of sorafenib-induced HFSR through the use of a murine model and cellular assays. By addressing this gap, the present work aims to provide a deeper mechanistic insight and to explore potential therapeutic avenues that could mitigate the adverse dermatological effects associated with sorafenib therapy.

## Materials and methods

### Cell lines and animals

Human immortalized keratinocytes (HaCaT) were obtained from Fenghui Biotechnology Co., Ltd. Cells were cultured in Dulbecco's Modified Eagle Medium (DMEM) supplemented with 10 % fetal bovine serum (FBS) at 37 °C in a 5 % CO_2_ humidified incubator. For in vivo experiments, six-week-old female Institute of Cancer Research (ICR) mice were purchased from Beijing Spebio Biotechnology Co., Ltd. Mice were housed under Specific Pathogen-Free (SPF) conditions with a 12-hour light/dark cycle, a temperature range of 18‒29 °C, and a relative humidity of 45 %‒55 %. The facility maintained a ventilation rate exceeding 10 air changes per hour. Animals had ad libitum access to standard rodent chow and water.

The study was approved by the Ethics Committee of the Affiliated Hospital of North Sichuan Medical College (NCMC-2022–048) and was conducted in accordance with the ARRIVE guidelines. All procedures also adhered to the ethical guidelines of the Helsinki Declaration.

### Drugs and reagents

Sorafenib (CAS: 284,461–73–0, Sigma, USA), the Cell Counting Kit-8 (CCK-8) assay kit (MA0218, Biosharp, China), penicillin-streptomycin (SV30010, Hyclone, USA), phosphate-buffered saline (PBS) (02–024–1ACS, VivaCell, China), trypsin (Hyclone, USA), formaldehyde fixative (Biosharp, China), EREG antibody (SAB, USA), EDTA decalcifying solution (E1171, Solarbio, China), recombinant EREG (X10507051, PrimeGene, China), FBS (1566,362, Gibco, USA), and DMEM (8120,502, Gibco, USA) were used in the experimental procedures.

### Drug preparation and HFSR model establishment

Sorafenib tablets were ground into a fine powder and mixed with a small volume of physiological saline to create a uniform suspension. This solution was prepared daily to the required concentration for oral administration. The experimental group (8 mice) received daily oral doses of sorafenib at 100 mg/kg body weight, corresponding to 0.1 mL per 10 g of body weight. The control group (8 mice) received an equal volume of physiological saline. Photographs of the paw conditions were taken on the day of grouping, and treatment continued daily thereafter. Mice were monitored for signs of paw swelling, erythema, and desquamation at five-day intervals, with images recorded. On day 30, mice were euthanized, and paw tissue samples were collected for further analysis.

### Histopathological observation

Paw tissue samples were fixed in 4 % formalin for 24 h, followed by rinsing with PBS and decalcification in EDTA solution for 10‒14 days. The tissues were dehydrated using an automated dehydration machine, cleared in xylene, and embedded in paraffin. Sections (4 µm thick) were prepared and stained with Hematoxylin and Eosin (H&E). A detailed histological evaluation was performed according to established criteria,^15^ including assessments of epidermal thickness, hyperkeratosis, inflammatory cell infiltration, and tissue architecture disruption. Each parameter was semi-quantitatively scored on a scale of 0 (no change) to 4 (severe alteration). Pathological changes were observed and documented under a light microscope.

### Immunohistochemistry staining

Paraffin-embedded sections were dewaxed, and antigen retrieval was performed using the standard protocol appropriate for the targeted antigen. Sections were incubated with primary antibodies against EREG (SAB, USA), followed by the appropriate secondary antibodies. DAB chromogen was applied for visual detection, and hematoxylin was used for nuclear counterstaining. After mounting, three representative images per sample were captured at 400× magnification. Image-Pro Plus 6.0 software was used to quantify the immunostaining by calculating the Integrated Optical Density (IOD) and tissue pixel area (AREA), with the Average Optical Density (AOD) determined using the formula AOD = IOD/AREA.

### CCK-8 assay

HaCaT cells were seeded in 96-well plates at a density of approximately 5 × 10³ cells per well in 100 µL of culture medium and allowed to adhere for 4 h at 37 °C. Cells were then treated with varying concentrations of sorafenib (5, 2.5, 1.25, 0.625 µmoL/L) and recombinant EREG (100 ng/mL, 50 ng/mL, 25 ng/mL). Each concentration was tested in six independent replicates, and each experiment was repeated independently three times. After 72 h of treatment, 10 µL of CCK-8 solution was added to each well, and the absorbance was measured at 450 nm after a further 2-hour incubation.

### Statistical analysis

Statistical analyses were performed using SPSS 19.0 software. Data are expressed as mean ± Standard Deviation (SD). Intergroup comparisons were performed using *t*-tests, with a significance threshold of *p* < 0.05.

## Results

### Construction of sorafenib-induced HFSR mouse model

**Fig. 1:**
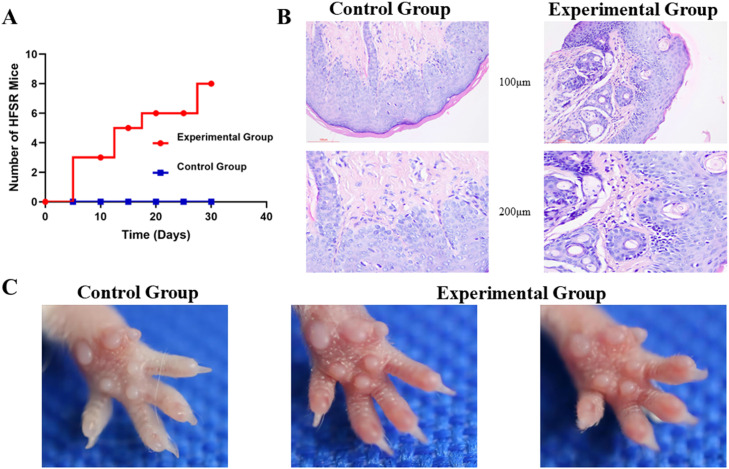
**Sorafenib-induced Hand-Foot Skin Reaction (HFSR) model in mice.** (A) Progression of HFSR symptoms in sorafenib-treated mice; (B) Histological examination of paw skin tissues in control and experimental group; (C) Comparison of paw skin in control and experimental group.

To establish a mouse model of sorafenib-induced HFSR, female ICR mice were administered sorafenib orally. After 10-days of treatment, erythema and swelling were observed in three out of eight mice. By day 30, all remaining mice exhibited pronounced HFSR symptoms, including red, dry, and swollen paws (Fig. 1A). Histopathological examination of the paw tissue revealed significant inflammatory cell infiltration predominantly characterized by neutrophils (Fig. 1B), consistent with the inflammatory profile observed in patients with HFSR.^16^ Notably, Fig. 1B also suggests an increase in sweat gland density in the affected areas. Although the pathophysiological relevance of this finding is not fully clear, it could reflect a compensatory hyperplastic response to local tissue injury. Mice that showed early symptoms of erythema and swelling later developed red, dry paws, closely resembling the clinical manifestations of HFSR in humans (Fig. 1C). In contrast, mice receiving physiological saline displayed no detectable skin lesions or inflammatory changes (Fig. 1B and C). These data demonstrate that the established sorafenib-induced HFSR mouse model closely resembles key clinical manifestations observed in human HFSR and is suitable for subsequent studies.

### Keratin layer thickening and elevated EREG expression in sorafenib-induced HFSR mouse model

**Fig. 2:**
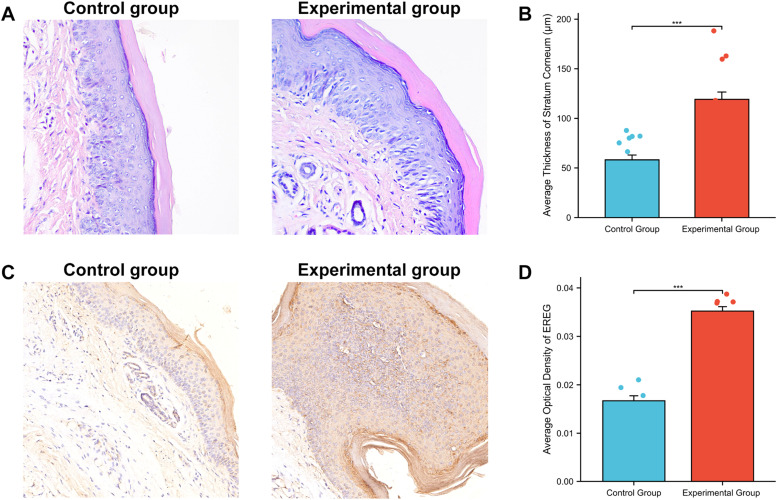
**Histological analysis of epidermal thickening and EREG expression in Hand-Foot Skin Reaction (HFSR) model.** (A) Representative hematoxylin and eosin staining in two groups of mice; (B) Average stratum corneum thickness in two groups of mice; (C) Representative immunohistochemistry staining of EREG in two groups of mice; (D) Average optical density of EREG in two groups of mice. * *p* < 0.05; ** *p* < 0.01, *** *p* < 0.001.

Histological analyses were performed to characterize the pathological changes associated with sorafenib-induced HFSR. Paw skin tissues from HFSR mice were subjected to H&E staining, and keratin layer thickness was quantified using Image-Pro Plus 6.0 software. The keratin layer thickness in the experimental group ranged from 88 to 188 µm, significantly higher than that observed in the control group (35‒87 µm, *p* < 0.001, Fig. 2A and B).

Additionally, immunohistochemical analysis revealed a statistically significant increase in EREG expression in the experimental group compared to the control group (*p* < 0.001, Fig. 2C and D). These findings suggest that elevated EREG expression is a characteristic feature of sorafenib-induced HFSR and may contribute to the pathological changes.

### EREG enhances keratinocyte proliferation

**Fig. 3:**
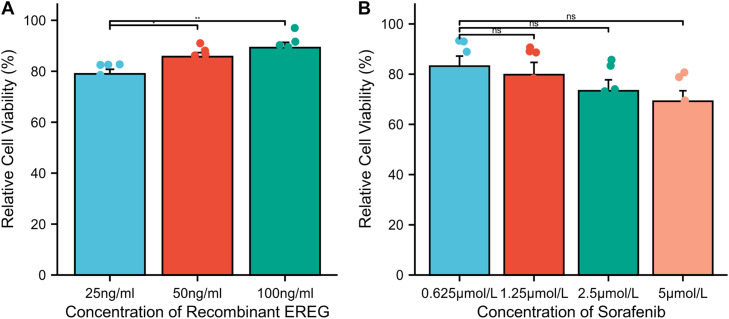
**Effects of sorafenib and recombinant EREG on HaCaT cell proliferation.** (A) Effect of different sorafenib concentration on HaCaT cell viability; (B) Effect of different recombinant EREG concentration on HaCaT cell viability. Ns, Not significant, * *p* < 0.05; ** *p* < 0.01.

To further evaluate the possible role of EREG in keratinocyte proliferation, HaCaT cells were treated with varying concentrations of recombinant EREG and sorafenib. Cell proliferation assays demonstrated that EREG significantly increased HaCaT cell proliferation in a dose-dependent manner, with 50 ng/mL and 100 ng/mL leading to statistically significant enhancements compared to 25 ng/mL (*p* < 0.05; *p* < 0.01, respectively; Fig. 3A). In contrast, sorafenib did not induce keratinocyte proliferation, as indicated by the lack of significant changes in HaCaT cell growth following different concentrations of sorafenib (Fig. 3B). These results suggest that while sorafenib does not directly stimulate keratinocyte proliferation, its administration leads to the upregulation of EREG, which in turn promotes keratinocyte proliferation and contributes to the thickening of the stratum corneum in HFSR. It is also plausible that sorafenib exerts indirect effects by modulating other inflammatory mediators and cytokine networks, thereby enhancing the overall cutaneous response.

## Discussion

The present study elucidates a potential mechanistic role for EREG in the pathogenesis of HFSR. The present findings demonstrated significant epidermal alterations in the murine model, with marked inflammatory cell infiltration predominantly characterized by neutrophils, and a substantial increase in keratin layer thickness that mirrors clinical observations in human HFSR. In vitro, while sorafenib did not induce keratinocyte proliferation, recombinant EREG significantly increased the proliferation of HaCaT cells, indicating that sorafenib-induced upregulation of EREG plays a central role in promoting keratinocyte hyperproliferation.

Histological analyses of the paw tissues from the HFSR mouse model revealed significant thickening of the keratin layer in the experimental group compared to controls (*p* < 0.001), consistent with findings in human patients.^16^ The increased keratin layer thickness is a hallmark of sorafenib-induced HFSR and highlights the drug’s impact on epidermal structure (Fig. 2A and B). Clinical evidence also suggested that interventions such as urea cream, which reduces keratinocyte density and induces epidermal thinning, can lower the incidence of severe sorafenib-induced HFSR in HCC patients.^17^, ^18^, ^19^

The use of a 100 mg/kg dose of sorafenib in the murine model warrants discussion. This dosage was selected based on Body Surface Area (BSA) normalization principles, as mice have higher metabolic rates and faster drug clearance than humans. FDA guidelines indicate that a human dose of 10 mg/kg is equivalent to approximately 123 mg/kg in mice when adjusted by a conversion factor of 12.3.^20^^,^^21^ The chosen dose of 100 mg/kg is slightly lower than this value, which may help balance efficacy and safety in the preclinical setting, while still producing the desired HFSR phenotype within the experimental timeframe.^22^

Several studies indicated that abnormal changes in keratinocytes are associated with various skin disorders, and EREG plays a role in keratinocyte proliferation and differentiation.^23^^,^^24^ As a member of the EGF family and an autocrine factor in normal human keratinocytes,^25^ the authors explored the expression of EREG in HFSR using immunohistochemistry staining. The results revealed that EREG expression was significantly higher in the sorafenib group compared to the control group (*p* < 0.001). Sorafenib is known to target Raf kinases, but recent studies reveal its broader effects. Notably, it inhibits MEK5-mediated activation of ERK5, a parallel branch of the MAPK cascade, and thus can alter EGF‑responsive gene expression.^26^^,^^27^ Moreover, oncogenic activation of the MEK/ERK pathway is a key driver of EREG expression in non-small-cell lung cancers.^28^ Consistently, treatment with ERK inhibitors blocks EREG‑dependent tumor-promoting effects in colorectal and lung cancer models, validating downstream ERK as an effector of EREG signaling.^29^ In addition, cell proliferation assays provided additional insights into the role of EREG in HFSR pathogenesis. The results demonstrated that EREG stimulation significantly enhanced the proliferation of HaCaT cells, a human keratinocyte cell line (Fig. 3). However, sorafenib did not directly promote keratinocyte proliferation, as corroborated by a recent study.^30^ These findings indicate that the upregulation of EREG in response to sorafenib may mediate keratinocyte proliferation, contributing to the excessive keratinization observed in HFSR.

Sorafenib is known as a multi-TKI that promotes apoptosis, reduces angiogenesis, and inhibits tumor cell proliferation. Research on how multi-TKIs lead to HFSR is limited. Some scholars suggested that HFSR may result from drug leakage following vascular damage or direct receptor action on exocrine glands.^6^^,^^31^ However, these theories do not fully explain the distinctive pathological features observed in HFSR, which is characterized by inflammatory cell infiltration and a thickened stratum corneum. Based on previous studies,^32^, ^33^, ^34^ the authors propose that sorafenib may cause local capillary dysregulation by targeting PDGFR-β and VEGFR-2, leading to epidermal ischemia and hypoxia. This local stress may trigger chronic inflammation and high EREG expression, which in turn promotes keratinocyte proliferation and thickening of the stratum corneum.

Despite the promise of EGFR pathway blockade, its application has been limited by on-target toxicities such as dermatologic reactions, gastrointestinal disturbances, and hepatotoxicity.^35^, ^36^, ^37^ These adverse events highlight the need for more selective approaches. Targeting the EGF-family ligand EREG using antibody-drug conjugates or neutralizing antibodies offers tumor specificity, as EREG is highly expressed in colorectal and lung cancers with low expression in normal tissues.^28^^,^^35^ Recent preclinical validation demonstrates potent antitumor efficacy with minimal toxicity in RAS mutant and wild-type models.^35^ Moreover, high tumor EREG expression predicts a favorable response to cetuximab in KRAS wild-type colorectal cancer, suggesting its utility as a companion biomarker.^36^ Clinical translation would involve patient selection based on EREG expression, rigorous safety profiling in early-phase trials, and possible combination with EGFR pathway inhibitors. Ultimately, direct EREG targeting could overcome resistance mechanisms while minimizing systemic EGFR-associated toxicity.

In addition, several important translational challenges must be acknowledged. First, EREG is physiologically expressed in keratinocytes and tissue-resident macrophages, where it plays key roles in skin barrier maintenance and wound healing.^11^^,^^13^ Therefore, systemic EREG inhibition could theoretically impair epidermal repair processes and delay cutaneous barrier recovery. Second, blockade of a single EGFR ligand may lead to compensatory upregulation of other ligands such as amphiregulin or HB-EGF, potentially sustaining downstream ERK signaling and reducing therapeutic efficacy.^23^^,^^29^ Third, clinical experience with cetuximab and other EGFR-targeted therapies has revealed characteristic toxicities, including dermatologic and gastrointestinal adverse events.^6^^,^^37^ Nevertheless, the efficacy of cetuximab in colorectal cancer illustrates that modulation of this pathway remains clinically feasible when carefully managed.^36^ Importantly, localized delivery of EGFR or EREG inhibitors may mitigate systemic toxicity, making such approaches attractive for conditions such as HFSR. Finally, emerging biotherapeutics, including EREG-specific neutralizing antibodies and antibody-drug conjugates, have shown robust antitumor efficacy with minimal toxicity in preclinical models.^35^ In the dermatologic setting, these modalities, particularly when formulated for topical application, may provide selective inhibition of EREG-driven keratinocyte proliferation while sparing systemic EGFR functions.

While this study provides insights into the mechanisms of sorafenib-induced HFSR, several limitations should be acknowledged. First, the use of a 100 mg/kg dose in mice, while necessary to overcome interspecies pharmacokinetic differences, does not directly correspond to the approximately 10 mg/kg human dose. Future studies should employ dose-scaling strategies or alternative models to better mimic human pharmacodynamics. Second, these experiments predominantly utilized animal models and in vitro assays, which may not fully capture the complexity of the clinical syndrome. Future research should aim to validate these findings in large-scale, multicenter clinical trials. Finally, while the present study identifies EREG as a key mediator of keratinocyte proliferation, further investigations are needed to elucidate the precise molecular pathways involved and to identify additional regulatory factors.

## Conclusion

In conclusion, the present study demonstrated that the upregulated EREG in sorafenib-induced HFSR may stimulate the proliferation of keratinocytes, leading to significant thickening of the stratum corneum. These findings provide a foundation for further research into the detailed mechanisms and potential interventions of sorafenib-induced HFSR.

## Funding

This study was supported by the National Natural Science Foundation of China (81402444) and, Natural Science Foundation of Sichuan Province (24NSFSC2149).

## Authors’ contributions

Y.L., X.H., and X.Z. were responsible for study conceptualization, experimental operation, data analysis, drafting and revision of the manuscript. Z.L. and X.H. were responsible for study conceptualization, validation, and revision of the manuscript. K.C., J.W., J.Z., Z.L., J.W., and G.Z. were responsible for validation and revision of the manuscript. All authors contributed to the article and approved the submitted version.

## Ethics approval and consent to participate

The study was approved by the Ethics Committee of the Affiliated Hospital of North Sichuan Medical College (NCMC-2022–048) and was conducted in accordance with the ARRIVE guidelines. All procedures also adhered to the ethical guidelines of the Helsinki Declaration.

## Informed consent and patient details

Not applicable.

## Declaration of competing interest

The authors declare no conflicts of interest.

## Data Availability

The data supporting the findings of this study are available from the corresponding author under reasonable requirements.
